# Analysis of the Most-Cited Systematic Review or Meta-Analysis in Acupuncture Research

**DOI:** 10.1155/2021/3469122

**Published:** 2021-09-16

**Authors:** Ying He, Yuxi Li, Juan Li, Ning Li, Yonggang Zhang, Nian Li

**Affiliations:** ^1^Department of Integrated Traditional and Western Medicine, West China Hospital, Sichuan University, Chengdu, China; ^2^School of Acupuncture-Moxibustion and Tuina, Chengdu University of Traditional Chinese Medicine, Chengdu, China; ^3^School of Health Preservation and Rehabilitation, Traditional Chinese Medicine of Chengdu University, Chengdu, Sichuan, China; ^4^Department of Periodical Press, West China Hospital, Sichuan University, Chengdu, China; ^5^Chinese Evidence-Based Medicine Center, West China Hospital, Sichuan University, Chengdu, China; ^6^Department of Medical Administration, West China Hospital, Sichuan University, Chengdu, China

## Abstract

**Objectives:**

The aim of the current study was to analyze the 100 most-cited systematic reviews or meta-analyses in the field of acupuncture research.

**Methods:**

The Web of Science Core Collection was used to retrieve lists of 100 most-cited systematic reviews or meta-analyses in the field of acupuncture research. Two authors screened literature, extracted data, and analyzed the results.

**Results:**

The citation number of the 100 most-cited systematic reviews or meta-analyses varied from 65 to 577; they were published between 1989 and 2018. Fourteen authors published more than 1 study as the corresponding author and 10 authors published more than 1 study as the first author. In terms of the corresponding authors, Edzard Ernst and Linde Klaus published the most systematic reviews/meta-analyses (*n* = 7). The USA published most of the systematic reviews or meta-analyses (*n* = 24), followed by England (*n* = 23) and China (*n* = 14). Most institutions with more than 1 study were from England (4/13). The institutions with the largest numbers of most-cited systematic reviews or meta-analyses were the Technical University of Munich in Germany, the University of Maryland School of Medicine in the USA (*n* = 8), the Universities of Exeter and Plymouth in England (*n* = 6), and the University of Exeter in England (*n* = 6). The journal with the largest number of most-cited systematic reviews or meta-analyses was the Cochrane Database of Systematic Reviews (*n* = 20), followed by Pain (*n* = 6).

**Conclusion:**

Our study reveals that the 100 most-cited systematic reviews or meta-analyses in the acupuncture research field are mostly from high impact factor journals and developed countries. It will help researchers follow research hot spots, broaden their research scope, expand their academic horizons, and explore new research ideas, thereby improving the quality of acupuncture research.

## 1. Introduction

Acupuncture is an important therapy in the traditional medicine of China and has been practiced for thousands of years [1]. It is defined as the insertion of one or several needles into the skin at particular sites for therapeutic purposes [1, 2]. The acupuncture research has shown impressive development during the past decade, and published articles have reflected its evolution [3, 4].

The Science Citation Index was initiated at the Institute for Scientific Information by Eugene Garfield, which is now owned by Clarivate Analytics [5]. It maintains a systematic ongoing measurement of citation counts for scientific journals and articles. The citation number of an article by other articles is widely considered as an important parameter to measure its relevance in the field of knowledge [6, 7]. Citation analysis can be used to identify studies that have influenced a given field, and a highly cited paper is usually seen as a landmark in any medical discipline, and it may influence further research and clinical practice [8]. In addition, the top-cited articles are often written by leading experts who can offer insight into future directions of the discipline and indicate the growth of particular fields [9].

Several recent studies have identified and analyzed citation classics and most-cited articles in various medical fields including gastric diseases [8], antibiotics [10], obstetrics and gynecology [7], and attention-deficit/hyperactivity disorder [11]. However, to the best of our knowledge, no comprehensive studies reported the most-cited systematic reviews or meta-analyses in acupuncture research, especially using the Web of Science database. Thus, we performed the current study to identify the 100 most-cited systematic reviews or meta-analyses published in journals in the field of acupuncture research.

## 2. Materials and Methods

### 2.1. Search Strategy

The Web of Science Core Collection was searched for the 100 most-cited systematic reviews/meta-analyses using the following search terms: (Acupuncture [Topic] OR Acupuncture [Title]) AND (systematic review [Topic] OR meta-analysis [Topic] OR systematic review [Title] OR meta-analysis [Title]). The last search was performed on May 5, 2021. The results were screened and ordered by the total number of citations, and the 100 most-cited systematic reviews or meta-analyses were included for data analysis.

### 2.2. Data Collection and Analysis

The 100 most-cited systematic reviews or meta-analyses were exported into one electronic datasheet and analyzed. Data were inserted into an Excel database (Excel 2010, Microsoft), and a descriptive statistical analysis was performed by Excel software. Structural visualization and analysis of the 100 most-cited studies were performed using VOSviewer 1.6.66 (https://www.vosviewer.com/, Leiden University Centre for Science and Technology Studies). For each study, the characteristics of the study such as the number of citations, ranking, authorship, title, year of publication, published journal, publication type, and topic categories were assessed by two authors. If the authors of an article had more than one affiliation, the department, institution, and country of origin were defined by the first affiliation of the first author.

## 3. Results

The search results included more than 1,500 systematic reviews or meta-analyses. After screening the titles and abstracts, the 100 most-cited systematic reviews or meta-analyses were selected for data analysis.  Table 1 shows the 100 most-cited systematic reviews or meta-analyses; they are ranked according to the number of citations. The total citation numbers of all 100 most-cited systematic reviews or meta-analyses were 13,459, varying from 65 to 577. Fifteen systematic reviews or meta-analyses had been cited more than 200 times. The most-cited systematic review/meta-analysis was Acupuncture, which was written by Vickers et al. in Arch. Intern. Med. published in 2012 with the citation number of 577 [12]. The second systematic review/meta-analysis was “Do certain countries produce only positive results? A systematic review of controlled trials” written by Vickers et al. published in Control Clin Trials in 1998 with the citation number of 425 [13]. The third systematic review/meta-analysis was “Is acupuncture effective for the treatment of chronic pain? A systematic review” written by Ezzo et al. published in Pain in 2000 with the citation number of 293 [14].

As to the authors, the results showed that there were 14 corresponding authors who published more than one study and 10 authors who published more than one study as first authors. In terms of the corresponding authors, Edzard Ernst and Linde Klaus published the most studies (*n* = 7). The author with most studies as the first author was Edzard Ernst who published 6 studies.  Table 2 summarised the characteristics of the authors.

As shown in Table 3, the 100 most-cited systematic reviews or meta-analyses were from 16 countries, respectively. The USA ranked first with 24 studies, followed by England (*n* = 23) and China (*n* = 14). When considering the average citation, the average citations of the Chinese authors were less than authors from other countries.

Table 4 shows institutions that published at least two most-cited studies. There were four institutions from England that published at least two most-cited studies, including the Universities of Exeter and Plymouth, the University of Exeter, the University of York, and Research Council for Complementary Medicine. As for the number of most-cited studies, the Technical University of Munich in Germany and the University of Maryland School of Medicine in the USA all ranked the first with eight studies; the Universities of Exeter and Plymouth in England ranked the second with seven studies.

Table 5 shows the published year of the 100 most-cited systematic reviews or meta-analyses. Most of these studies were published in 2009 (*n* = 9) and 2010 (*n* = 9), and 8 studies were published in 2005, 2008, and 2013.

The 100 most-cited systematic reviews or meta-analyses were published in 50 journals (Table 6). Two systematic reviews or meta-analyses were from stroke and were cited with an average of 133 times, which was in the moderate level. Among the 50 journals, impact factors (2020) of 5 journals were higher than 10, including Annals of Internal Medicine, British Journal of Sports Medicine, British Medical Journal, Journal of Clinical Oncology, and JAMA Internal Medicine.

**Figure 1:**
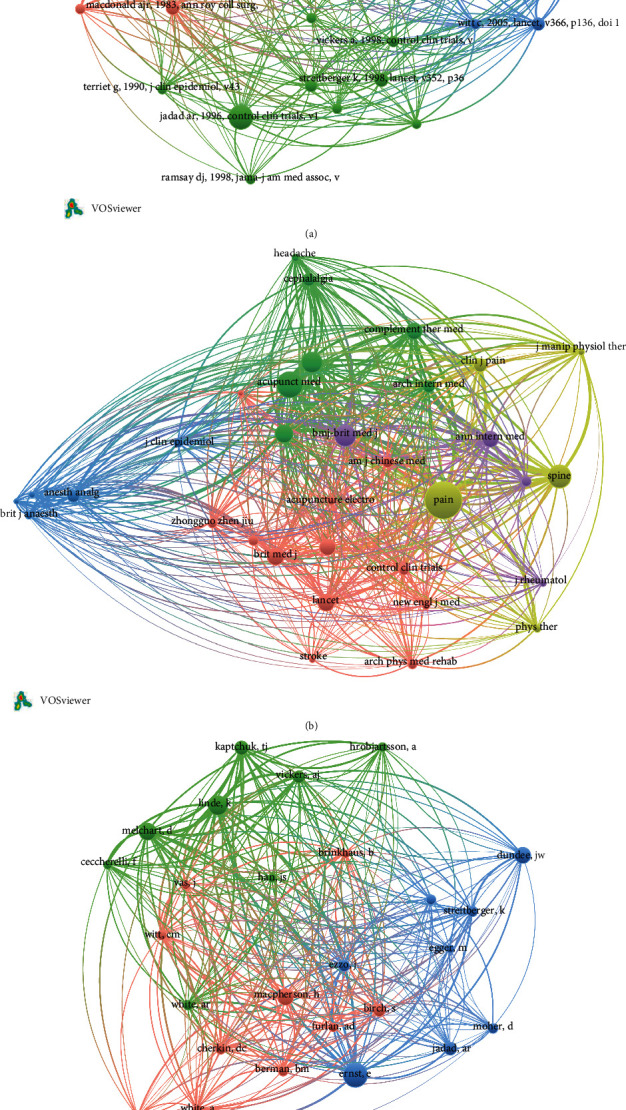
The cocitation between the included studies. (a) The most frequent cocitation reference: Studies by Controlled Clinical Trials, British Medical Journal, and Annals of Internal Medicine were the most frequently cocitation references. (b) The most frequent cocitation source: Studies from Pain, Acupuncture in Medicine, Spine, and British Medical Journal were the most frequently cocitation source. (c) The most frequent cocitation author. Studies by Edzard Ernst, Linde Klaus, MacPherson, and Hugh are the most frequently cocitation author.

The cocitation of the 100 top-cited studies is shown in Figure 1. The most frequent cocited study was about quantifying heterogeneity in a meta-analysis (*n* = 31) published by Controlled Clinical Trials in 1996. The most frequent cocitied source was Pain (*n* = 280). Edzard Ernst from the University of Exeter in England was the most frequent cocitied author (*n* = 70).

## 4. Discussion

Acupuncture is undoubtedly valuable in Chinese traditional medicine. It is effective, especially in reducing chronic pain, such as cervical spondylosis and the management of low back pain [15–17]. In the field of acupuncture research, citation of studies varied. The most-cited studies ranked the highest position and could show research ability and development in different countries and institutions. Thus, analyzing the most-cited studies would help future studies.

The numbers of citations of the 100 most-cited systematic reviews or meta-analyses were considerable. The data indicated that the researchers in the medical field give huge attention to acupuncture. Our results showed the top researches of acupuncture, investigating the distribution of authors, countries, institutions, year, and journals. The study showed that authors from the USA published most studies among the 100 most-cited studies. Although China is the birth land of acupuncture, the number of Chinese studies was only 14. Traditional Chinese clinicians might always focus on the clinical practice, ignoring the research, especially the study using modern scientific methods [18]. On the contrary, western researchers were proficient in research methods. So when they develop great interests in this field, they could perform studies with high quality.

The institutions revealed that researchers in famous research-based universities or hospitals made huge efforts to investigate the principle and effectiveness of acupuncture, such as Technische Universität München, Harvard University, Massachusetts General Hospital, and the University of Maryland School of Medicine. Acupuncture was the traditional therapeutic method with large varieties of clinical practice, especially in China and other East Asian countries, which suggest that more attention should be paid to China in international journals.

In terms of journals, the number of systematic reviews or meta-analyses from the Cochrane Database of Systematic Reviews was the largest (*n* = 20) because this journal is dedicated to publishing systematic reviews. Besides, some highly influential journals, such as Annals of Internal Medicine, BMJ, and Journal of Clinical Oncology, also published some studies in this field. It is undoubted that papers published in highly influential journals would be cited more because of the great reputation of these journals; thus, we should publish more studies in highly influential journals.

In terms of the cocitation of the 100 most-cited studies, the results indicated that the most-cited studies were published in Pain; this might be related to the limitation of understanding on acupuncture by foreign researchers. Hence, it suggested that editors and authors should choose other interesting research topics of studies in the future.

It should be noted that Chinese researchers contributed 14 papers, and some Chinese authors were really outstanding. Jian-Ping Liu from Beijing University of Chinese Medicine published 1 study in PLoS One [19] and Journal of Alternative and Complementary Medicine [20]. Lee Anna from the Chinese University of Hong Kong published 2 studies in the Cochrane Database of Systematic Reviews [21, 22]. Sze, FKH from Shatin Hospital in Hong Kong published 1 study in Stroke [23], while Huang Qiang-Min from Shanghai University of Sports in Shanghai published 1 study in Archives of Physical Medicine and Rehabilitation [24]. It should be noted that acupuncture was used most widely in China; due to language constraints, some excellent Chinese studies might not be cited, which might bias the conclusion of this study. The strengthening of exchanges and cooperation with international institutions and institutions had been identified as a priority in the acupuncture field. Hence, the acupuncture researchers should pay attention to the research in high-impact institutions and published high-impact researches in a timely manner, in order to promote interinstitutional cooperation and exchanges.

There were some limitations of our study. Our electronic search was performed in the Web of Science Core Collection. As a result, the bias of searching cannot be avoided [25]. In addition, a number of studies conducted by Chinese researchers were published in Chinese journals and have not been included in the current study, so the results should be explained with caution.

In conclusion, our study revealed that the 100 most-cited systematic reviews or meta-analyses in the acupuncture research field are mostly from high impact factors journals and are mostly from western counties. It will help researchers follow research hot spots, broaden their research scope, expand their academic horizons, and explore new research ideas, thereby improving the quality of acupuncture research.

## Figures and Tables

**Table 1:** The 100 most-cited acupuncture research works.

Ranking	Article	Disease	Citation
1	Vickers AJ, Cronin AM, Maschino AC, et al. Acupuncture for chronic pain: individual patient data meta-analysis. Arch Intern Med. 2012; 172(19):1444–1453.	Chronic pain	577
2	Vickers A, Goyal N, Harland R, Rees R. Do certain countries produce only positive results? A systematic review of controlled trials. Control Clin Trials. 1998; 19(2):159–166.	Controlled trials	425
3	Ezzo J, Berman B, Hadhazy VA, Jadad AR, Lao L, Singh BB. Is acupuncture effective for the treatment of chronic pain? A systematic review. Pain. 2000; 86(3):217–225.	Chronic pain	293
4	Huang W, Pach D, Napadow V, et al. Characterizing acupuncture stimuli using brain imaging with FMRI--a systematic review and meta-analysis of the literature. PLoS One. 2012; 7(4):e32960.	Brain imaging with fMRI	272
5	Manheimer E, White A, Berman B, Forys K, Ernst E. Meta-analysis: acupuncture for low back pain [published correction appears in Ann Intern Med. 2005 Jun 7; 142(11):950–1]. Ann Intern Med. 2005; 142(8):651–663.	Low back pain	261
6	Cherkin DC, Sherman KJ, Deyo RA, Shekelle PG. A review of the evidence for the effectiveness, safety, and cost of acupuncture, massage therapy, and spinal manipulation for back pain. Ann Intern Med. 2003; 138(11):898–906.	Back pain	252
7	Furlan AD, van Tulder MW, Cherkin DC, et al. Acupuncture and dry-needling for low back pain. Cochrane Database Syst Rev. 2005; (1):CD001351. Published 2005 Jan 25.	Low back pain	246
8	Cummings TM, White AR. Needling therapies in the management of myofascial trigger point pain: a systematic review. Arch Phys Med Rehabil. 2001; 82(7):986–992.	Myofascial trigger point pain	238
9	Hróbjartsson A, Gøtzsche PC. Is the placebo powerless? Update of a systematic review with 52 new randomized trials comparing placebo with no treatment. J Intern Med. 2004; 256(2):91–100.	Randomized trials	237
10	Green S, Buchbinder R, Hetrick S. Acupuncture for shoulder pain. Cochrane Database Syst Rev. 2005; (2):CD005319. Published 2005 Apr 18.	Shoulder pain	230
11	Chen W, Yang GY, Liu B, Manheimer E, Liu JP. Manual acupuncture for treatment of diabetic peripheral neuropathy: a systematic review of randomized controlled trials [published correction appears in PLoS One. 2014; 9(3):e91110]. PLoS One. 2013; 8(9):e73764. Published 2013 Sep 12.	Diabetic peripheral neuropathy	220
12	Linde K, Allais G, Brinkhaus B, Manheimer E, Vickers A, White AR. Acupuncture for migraine prophylaxis. Cochrane Database Syst Rev. 2009; (1):CD001218. Published 2009 Jan 21.	Migraine	213
13	Furlan AD, van Tulder M, Cherkin D, et al. Acupuncture and dry-needling for low back pain: an updated systematic review within the framework of the cochrane collaboration. Spine (Phila Pa 1976). 2005; 30(8):944–963.	Low back pain	206
14	Bjordal JM, Johnson MI, Ljunggreen AE. Transcutaneous electrical nerve stimulation (TENS) can reduce postoperative analgesic consumption. A meta-analysis with assessment of optimal treatment parameters for postoperative pain. Eur J Pain. 2003; 7(2):181–188.	Postoperative pain	203
15	Vickers AJ. Can acupuncture have specific effects on health? A systematic review of acupuncture antiemesis trials. J R Soc Med. 1996; 89(6):303–311.	Antiemesis	202
16	Bisset L, Paungmali A, Vicenzino B, Beller E. A systematic review and meta-analysis of clinical trials on physical interventions for lateral epicondylalgia. Br J Sports Med. 2005; 39(7):411–422.	Lateral epicondylalgia	198
17	Madsen MV, Gøtzsche PC, Hróbjartsson A. Acupuncture treatment for pain: systematic review of randomized clinical trials with acupuncture, placebo acupuncture, and no acupuncture groups. BMJ. 2009; 338:a3115. Published 2009 Jan 27.	Pain	195
18	Lee A, Done ML. The use of nonpharmacologic techniques to prevent postoperative nausea and vomiting: a meta-analysis. Anesth Analg. 1999; 88(6):1362–1369.	Postoperative nausea and vomiting	187
19	Bjordal JM, Johnson MI, Lopes-Martins RA, Bogen B, Chow R, Ljunggren AE. Short-term efficacy of physical interventions in osteoarthritic knee pain. A systematic review and meta-analysis of randomized placebo-controlled trials. BMC Musculoskelet Disord. 2007; 8:51. Published 2007 Jun 22.	Osteoarthritic knee pain	186
20	Ezzo J, Hadhazy V, Birch S, et al. Acupuncture for osteoarthritis of the knee: a systematic review. Arthritis Rheum. 2001; 44(4):819–825.	Osteoarthritis of the knee	171
21	Moffet HH. Sham acupuncture may be as efficacious as true acupuncture: a systematic review of clinical trials. J Altern Complement Med. 2009; 15(3):213–216.	Clinical trials	168
22	Tough EA, White AR, Cummings TM, Richards SH, Campbell JL. Acupuncture and dry needling in the management of myofascial trigger point pain: a systematic review and meta-analysis of randomized controlled trials. Eur J Pain. 2009; 13(1):3–10.	Myofascial trigger point pain	167
23	White A, Foster NE, Cummings M, Barlas P. Acupuncture treatment for chronic knee pain: a systematic review. Rheumatology (Oxford). 2007; 46(3):384–390.	Chronic knee pain	165
24	Garcia MK, McQuade J, Haddad R, et al. Systematic review of acupuncture in cancer care: a synthesis of the evidence. J Clin Oncol. 2013; 31(7):952–960.	Cancer care	163
25	Ernst E, White AR. Prospective studies of the safety of acupuncture: a systematic review. Am J Med. 2001; 110(6):481–485.	Safety of acupuncture	161
26	Sun Y, Gan TJ, Dubose JW, Habib AS. Acupuncture and related techniques for postoperative pain: a systematic review of randomized controlled trials. Br J Anesth. 2008; 101(2):151–160.	Postoperative pain	160
27	Ernst E. Acupuncture - a critical analysis. J Intern Med. 2006; 259(2):125–137.	A critical analysis	160
28	van Tulder MW, Cherkin DC, Berman B, Lao L, Koes BW. The effectiveness of acupuncture in the management of acute and chronic low back pain. A systematic review within the framework of the Cochrane Collaboration Back Review Group. Spine (Phila Pa 1976). 1999; 24(11):1113–1123.	Acute and chronic low back pain	160
29	Ernst E, White AR. Acupuncture for back pain: a meta-analysis of randomized controlled trials. Arch Intern Med. 1998; 158(20):2235–2241.	Back pain	159
30	Manheimer E, Cheng K, Linde K, et al. Acupuncture for peripheral joint osteoarthritis. Cochrane Database Syst Rev. 2010; (1):CD001977. Published 2010 Jan 20.	Peripheral joint osteoarthritis	151
31	Eric M, Klaus L, Lixing L, M BL, M BB. Meta-analysis: acupuncture for osteoarthritis of the knee. Ann Intern Med. 2007; 146(12).	Osteoarthritis of the knee	148
32	Smith LA, Oldman AD, McQuay HJ, Moore RA. Teasing apart quality and validity in systematic reviews: an example from acupuncture trials in chronic neck and back pain. Pain. 2000; 86(1–2):119–132.	Chronic neck and back pain	147
33	Vickers AJ, Vertosick EA, Lewith G, et al. Acupuncture for Chronic Pain: Update of an Individual Patient Data Meta-Analysis. J Pain. 2018; 19(5):455–474.	Chronic Pain	146
34	Furlan AD, Yazdi F, Tsertsvadze A, et al. A systematic review and meta-analysis of efficacy, cost-effectiveness, and safety of selected complementary and alternative medicine for neck and low-back pain. Evid Based Complement Alternat Med. 2012; 2012:953139.	Neck and low back pain	145
35	White AR, Ernst E. A systematic review of randomized controlled trials of acupuncture for neck pain. Rheumatology (Oxford). 1999; 38(2):143–147.	Neck pain	144
36	Meissner K, Fässler M, Rücker G, et al. Differential effectiveness of placebo treatments: a systematic review of migraine prophylaxis. JAMA Intern Med. 2013; 173(21):1941–1951.	Migraine	140
37	Geeganage C, Beavan J, Ellender S, Bath PM. Interventions for dysphagia and nutritional support in acute and subacute stroke. Cochrane Database Syst Rev. 2012; 10:CD000323. Published 2012 Oct 17.	Acute and subacute stroke	139
38	Ahn AC, Colbert AP, Anderson BJ, et al. Electrical properties of acupuncture points and meridians: a systematic review. Bioelectromagnetics. 2008; 29(4):245–256.	Acupuncture points and meridians	139
39	Johnson M, Martinson M. Efficacy of electrical nerve stimulation for chronic musculoskeletal pain: a meta-analysis of randomized controlled trials. Pain. 2007; 130(1–2):157–165.	Chronic musculoskeletal pain	137
40	Kietrys DM, Palombaro KM, Azzaretto E, et al. Effectiveness of dry needling for upper-quarter myofascial pain: a systematic review and meta-analysis. J Orthop Sports Phys Ther. 2013; 43(9):620–634.	Myofascial Pain	136
41	Melchart D, Linde K, Fischer P, et al. Acupuncture for recurrent headaches: a systematic review of randomized controlled trials [published correction appears in Cephalalgia 2000 Oct; 20(8):762–3]. Cephalalgia. 1999; 19(9):779–765.	Recurrent headaches	133
42	Khadilkar A, Odebiyi DO, Brosseau L, Wells GA. Transcutaneous electrical nerve stimulation (TENS) versus placebo for chronic low-back pain. Cochrane Database Syst Rev. 2008; 2008(4):CD003008. Published 2008 Oct 8.	Chronic low back pain	132
43	Wu P, Mills E, Moher D, Seely D. Acupuncture in poststroke rehabilitation: a systematic review and meta-analysis of randomized trials. Stroke. 2010; 41(4):e171-e179.	Poststroke rehabilitation	129
44	Lao L, Hamilton GR, Fu J, Berman BM. Is acupuncture safe? A systematic review of case reports. Altern Ther Health Med. 2003; 9(1):72–83.	Safe	122
45	Lee A, Fan LT. Stimulation of the wrist acupuncture point P6 for preventing postoperative nausea and vomiting. Cochrane Database Syst Rev. 2009; (2):CD003281. Published 2009 Apr 15.	Postoperative nausea and vomiting	120
46	Ernst E, White A. Life-threatening adverse reactions after acupuncture? A systematic review. Pain. 1997; 71(2):123–126.	Adverse reactions	120
47	Dincer F, Linde K. Sham interventions in randomized clinical trials of acupuncture--a review. Complement Ther Med. 2003; 11(4):235–242.	Sham interventions	117
48	Linde K, Niemann K, Schneider A, Meissner K. How large are the nonspecific effects of acupuncture? A meta-analysis of randomized controlled trials. BMC Med. 2010; 8:75. Published 2010 Nov 23.	The nonspecific effects of acupuncture	115
49	Manheimer E, Zhang G, Udoff L, et al. Effects of acupuncture on rates of pregnancy and live birth among women undergoing in vitro fertilisation: systematic review and meta-analysis. BMJ. 2008; 336(7643):545–549.	Pregnancy and live birth among women undergoing in vitro fertilisation	115
50	Zhang ZJ, Chen HY, Yip KC, Ng R, Wong VT. The effectiveness and safety of acupuncture therapy in depressive disorders: systematic review and meta-analysis. J Affect Disord. 2010; 124(1–2):9–21.	Depressive disorders	114
51	Prady SL, Richmond SJ, Morton VM, Macpherson H. A systematic evaluation of the impact of STRICTA and CONSORT recommendations on quality of reporting for acupuncture trials. PLoS One. 2008; 3(2):e1577. Published 2008 Feb 13.	Acupuncture trials	113
52	Linde K, Allais G, Brinkhaus B, Manheimer E, Vickers A, White AR. Acupuncture for tension-type headache. Cochrane Database Syst Rev. 2009; (1):CD007587. Published 2009 Jan 21.	Tension-type headache	110
53	Kalichman L, Vulfsons S. Dry needling in the management of musculoskeletal pain. J Am Board Fam Med. 2010; 23(5):640–646.	Musculoskeletal pain	109
54	Smith CA, Hay PP, Macpherson H. Acupuncture for depression. Cochrane Database Syst Rev. 2010; (1):CD004046. Published 2010 Jan 20.	Depression	106
55	Ezzo JM, Richardson MA, Vickers A, et al. Acupuncture-point stimulation for chemotherapy-induced nausea or vomiting. Cochrane Database Syst Rev. 2006; (2):CD002285. Published 2006 Apr 19.	Nausea or vomiting	106
56	Trinh KV, Graham N, Gross AR, et al. Acupuncture for neck disorders. Cochrane Database Syst Rev. 2006; (3):CD004870. Published 2006 Jul 19.	Neck disorders	106
57	Patel M, Gutzwiller F, Paccaud F, Marazzi A. A meta-analysis of acupuncture for chronic pain. Int J Epidemiol. 1989; 18(4):900–906.	Chronic pain	106
58	Yuan J, Purepong N, Kerr DP, Park J, Bradbury I, McDonough S. Effectiveness of acupuncture for low back pain: a systematic review. Spine (Phila Pa 1976). 2008; 33(23):E887-E900.	Low back pain	104
59	Jindal V, Ge A, Mansky PJ. Safety and efficacy of acupuncture in children: a review of the evidence. J Pediatr Hematol Oncol. 2008; 30(6):431–442.	Children	104
60	Ernst E, Lee MS, Choi TY. Acupuncture: does it alleviate pain and are there serious risks? A review of reviews. Pain. 2011; 152(4):755–764.	Alleviate pain	102
61	Trinh KV, Phillips SD, Ho E, Damsma K. Acupuncture for the alleviation of lateral epicondyle pain: a systematic review. Rheumatology (Oxford). 2004; 43(9):1085–1090.	Lateral epicondyle pain	102
62	Berman BM, Ezzo J, Hadhazy V, Swyers JP. Is acupuncture effective in the treatment of fibromyalgia?. J Fam Pract. 1999; 48(3):213–218.	Fibromyalgia	100
63	Sze FK, Wong E, Or KK, Lau J, Woo J. Does acupuncture improve motor recovery after stroke? A meta-analysis of randomized controlled trials. Stroke. 2002; 33(11):2604–2619.	Recovery after stroke	96
64	Linde K, Allais G, Brinkhaus B, et al. Acupuncture for the prevention of episodic migraine. Cochrane Database Syst Rev. 2016; 2016(6):CD001218. Published 2016 Jun 28.	Migraine	95
65	Liu L, Huang QM, Liu QG, et al. Effectiveness of dry needling for myofascial trigger points associated with neck and shoulder pain: a systematic review and meta-analysis. Arch Phys Med Rehabil. 2015; 96(5):944–955.	Neck and shoulder pain	94
66	White A, Cummings M, Barlas P, et al. Defining an adequate dose of acupuncture using a neurophysiological approach--a narrative review of the literature. Acupunct Med. 2008; 26(2):111–120.	Neurophysiological	93
67	Kwon YD, Pittler MH, Ernst E. Acupuncture for peripheral joint osteoarthritis: a systematic review and meta-analysis. Rheumatology (Oxford). 2006; 45(11):1331–1337.	Peripheral joint osteoarthritis	93
68	Asher GN, Jonas DE, Coeytaux RR, et al. Auriculotherapy for pain management: a systematic review and meta-analysis of randomized controlled trials. J Altern Complement Med. 2010; 16(10):1097–1108.	Pain	90
69	Cho SH, Lee JS, Thabane L, Lee J. Acupuncture for obesity: a systematic review and meta-analysis. Int J Obes (Lond). 2009; 33(2):183–196.	Obesity	89
70	Lim B, Manheimer E, Lao L, et al. Acupuncture for treatment of irritable bowel syndrome. Cochrane Database Syst Rev. 2006; (4):CD005111. Published 2006 Oct 18.	Irritable bowel syndrome	89
71	Ernst E, Pittler MH. The effectiveness of acupuncture in treating acute dental pain: a systematic review. Br Dent J. 1998; 184(9):443–447.	Acute dental pain	88
72	Lee A, Chan SK, Fan LT. Stimulation of the wrist acupuncture point PC6 for preventing postoperative nausea and vomiting. Cochrane Database Syst Rev. 2015; 2015(11):CD003281. Published 2015 Nov 2.	Postoperative nausea and vomiting	87
73	Cao H, Pan X, Li H, Liu J. Acupuncture for treatment of insomnia: a systematic review of randomized controlled trials. J Altern Complement Med. 2009; 15(11):1171–1186.	Insomnia	86
74	Casimiro L, Barnsley L, Brosseau L, et al. Acupuncture and electroacupuncture for the treatment of rheumatoid arthritis. Cochrane Database Syst Rev. 2005; (4):CD003788. Published 2005 Oct 19.	Rheumatoid arthritis	84
75	Park J, Hopwood V, White AR, Ernst E. Effectiveness of acupuncture for stroke: a systematic review. J Neurol. 2001; 248(7):558–563.	Stroke	84
76	Corbett MS, Rice SJ, Madurasinghe V, Slack R, Fayter DA, Harden M, Sutton AJ, Macpherson H, Woolacott NF. Acupuncture and other physical treatments for the relief of pain due to osteoarthritis of the knee: network meta-analysis. Osteoarthritis Cartilage. 2013 Sep; 21(9):1290–8.	Osteoarthritis of the knee	82
77	Zhang J, Shang H, Gao X, Ernst E. Acupuncture-related adverse events: a systematic review of the Chinese literature. Bull World Health Organ. 2010; 88(12):915–921C.	Adverse events	81
78	Adams D, Cheng F, Jou H, Aung S, Yasui Y, Vohra S. The safety of pediatric acupuncture: a systematic review. Pediatrics. 2011; 128(6):e1575-e1587.	Pediatric acupuncture	80
79	Yamashita H, Tsukayama H, White AR, Tanno Y, Sugishita C, Ernst E. Systematic review of adverse events following acupuncture: the Japanese literature. Complement Ther Med. 2001; 9(2):98–104.	Adverse events	75
80	Lee H, Schmidt K, Ernst E. Acupuncture for the relief of cancer-related pain--a systematic review. Eur J Pain. 2005; 9(4):437–444.	Cancer-related pain	74
81	Brosseau L, Milne S, Robinson V, et al. Efficacy of the transcutaneous electrical nerve stimulation for the treatment of chronic low back pain: a meta-analysis. Spine (Phila Pa 1976). 2002; 27(6):596–603.	Chronic low back pain	74
82	Linde K, Niemann K, Meissner K. Are sham acupuncture interventions more effective than (other) placebos? A re-analysis of data from the Cochrane review on placebo effects. Forsch Komplementmed. 2010; 17(5):259–264.	Placebo effects	73
83	Dowswell T, Bedwell C, Lavender T, Neilson JP. Transcutaneous electrical nerve stimulation (TENS) for pain relief in labour. Cochrane Database Syst Rev. 2009; (2):CD007214. Published 2009 Apr 15.	Pain relief in labour	73
84	Lam M, Galvin R, Curry P. Effectiveness of acupuncture for nonspecific chronic low back pain: a systematic review and meta-analysis. Spine (Phila Pa 1976). 2013; 38(24):2124–2138.	Nonspecific chronic low back pain	72
85	Dodin S, Blanchet C, Marc I, et al. Acupuncture for menopausal hot flushes. Cochrane Database Syst Rev. 2013; 2013(7):CD007410. Published 2013 Jul 30.	Menopausal hot flushes	71
86	Yeung WF, Chung KF, Poon MM, et al. Acupressure, reflexology, and auricular acupressure for insomnia: a systematic review of randomized controlled trials. Sleep Med. 2012; 13(8):971–984.	Insomnia	71
87	Mayhew E, Ernst E. Acupuncture for fibromyalgia--a systematic review of randomized clinical trials. Rheumatology (Oxford). 2007; 46(5):801–804.	Fibromyalgia	70
88	Manyanga T, Froese M, Zarychanski R, et al. Pain management with acupuncture in osteoarthritis: a systematic review and meta-analysis. BMC Complement Altern Med. 2014; 14:312. Published 2014 Aug 23.	Osteoarthritis	68
89	Cheuk DK, Yeung WF, Chung KF, Wong V. Acupuncture for insomnia. Cochrane Database Syst Rev. 2012; (9):CD005472. Published 2012 Sep 12.	Insomnia	68
90	Hurlow A, Bennett MI, Robb KA, Johnson MI, Simpson KH, Oxberry SG. Transcutaneous electric nerve stimulation (TENS) for cancer pain in adults. Cochrane Database Syst Rev. 2012; 2012(3):CD006276. Published 2012 Mar 14.	Cancer pain	68
91	Vas J, Perea-Milla E, Méndez C, et al. Efficacy and safety of acupuncture for chronic uncomplicated neck pain: a randomized controlled study. Pain. 2006; 126(1–3):245–255.	Chronic uncomplicated neck pain	67
92	Streitberger K, Ezzo J, Schneider A. Acupuncture for nausea and vomiting: an update of clinical and experimental studies. Auton Neurosci. 2006; 129(1–2):107–117.	Nausea and vomiting	67
93	Gattie E, Cleland JA, Snodgrass S. The Effectiveness of Trigger Point Dry Needling for Musculoskeletal Conditions by Physical Therapists: A Systematic Review and Meta-analysis. J Orthop Sports Phys Ther. 2017; 47(3):133–149.	Musculoskeletal conditions	66
94	Li H, He T, Xu Q, et al. Acupuncture and regulation of gastrointestinal function. World J Gastroenterol. 2015; 21(27):8304–8313.	Gastrointestinal function	66
95	Lee JH, Choi TY, Lee MS, Lee H, Shin BC, Lee H. Acupuncture for acute low back pain: a systematic review. Clin J Pain. 2013; 29(2):172–185.	Acute low back pain	66
96	Leo RJ, Ligot JS Jr. A systematic review of randomized controlled trials of acupuncture in the treatment of depression. J Affect Disord. 2007; 97(1–3):13–22.	Depression	66
97	Zhang SH, Liu M, Asplund K, Li L. Acupuncture for acute stroke. Cochrane Database Syst Rev. 2005; (2):CD003317. Published 2005 Apr 18.	Acute stroke	66
98	Wu MS, Chen KH, Chen IF, et al. The Efficacy of Acupuncture in Post-Operative Pain Management: A Systematic Review and Meta-Analysis. PLoS One. 2016; 11(3):e0150367. Published 2016 Mar 9.	Postoperative pain	65
99	Zhou J, Peng W, Xu M, Li W, Liu Z. The effectiveness and safety of acupuncture for patients with Alzheimer disease: a systematic review and meta-analysis of randomized controlled trials. Medicine (Baltimore). 2015; 94(22):e933.	Alzheimer's disease	65
100	MacPherson H, Maschino AC, Lewith G, et al. Characteristics of acupuncture treatment associated with outcome: an individual patient meta-analysis of 17,922 patients with chronic pain in randomized controlled trials [published correction appears in PLoS One. 2013; 8(12).	Chronic pain	65

**Table 2:** Authors with more than 1 study as first author or corresponding author included in the 100 most-cited studies.

Author	Name	Number of studies
Corresponding author	Ernst E	7
	Linde K	7
	Manheimer E	5
	Vickers AJ	3
	Lee A	3
	Hrobjartsson A	2
	Lee H	2
	Trinh KV	2
	White A	2
	Liu JP	2
	Furlan AD	2
	Ezzo J	2
	Brosseau, L	2
	Bjordal JM	2

First author	Ernst E	6
	Linde K	5
	Furlan AD	3
	Lee A	3
	Vickers AJ	3
	Manheimer E	3
	Trinh KV	2
	White A	2
	Bjordal JM	2
	Ezzo J	2

**Table 3:** Country of origin of the 100 most-cited studies (based on country of the first author).

Addresses	Number of studies	Total citations	Average citation	Number of studies in each ranking										
				50–99	100–149	150–199	200–249	250–299	300–349	350–399	400–449	450–499	500–549	550–599
USA	24	3,920	163	4	10	5		3						1
England	23	3,158	137	8	6	5	2				1			
China	14	1,299	93	11	2		1							
Canada	11	1,237	112	5	5		1							
Germany	10	1,335	134	3	5		1	1						
Australia	4	721	180		1	2	1							
Denmark	2	432	216			1	1							
Netherlands	1	206	206				1							
Norway	2	389	195			1	1							
South Korea	3	229	76	3										
Japan	1	75	75	1										
Ireland	1	72	72	1										
Spain	1	67	67	1										
Israel	1	109	109		1									
Switzerland	1	106	106		1									
North Ireland	1	104	104		1									

**Table 4:** Institutions with at least 2 studies based on the institution of the corresponding authors included in the 100 most-cited studies.

Country	Institution	Number of studies
Canada		8
	University of Ottawa	4
	McMaster University	2
	Institute for Work & Health	2

China		7
	Beijing University of Chinese Medicine	2
	University of Hong Kong	3
	Chinese University of Hong Kong	2

England		17
	Universities of Exeter and Plymouth	6
	University of Exeter	6
	The University of York	3
	Research Council for Complementary Medicine	2

Germany		10
	Technische Universität München	8
	Nordic Cochrane Centre	2

South Korea		2
	Kyung Hee University	2

USA		10
	University of Maryland School of Medicine	8
	Memorial Sloan Kettering Cancer Center	2

**Table 5:** Distribution by year of publication of the 100 most-cited studies.

Year	Number of study	Total citation	Average citation
1989	1	106	106
1996	1	202	202
1997	1	120	120
1998	3	672	224
1999	5	724	145
2000	2	440	220
2001	5	729	146
2002	2	170	85
2003	4	694	174
2004	2	339	170
2005	8	1,365	171
2006	7	688	103
2007	6	772	129
2008	8	960	120
2009	9	1,221	136
2010	9	968	108
2011	2	182	91
2012	7	1,340	192
2013	8	795	99
2014	2	288	144
2015	4	312	78
2016	2	160	80
2017	1	66	66
2018	1	146	146

**Table 6:** Journals in which the 100 most-cited studies were published.

Journal	Abbreviated name	Articles included in top 100 cited	Total citations	Average citation/article	Impact factor (2021)
Archives of Internal Medicine	Arch. Intern. Med.	2	736	368	NA
Annals of Internal Medicine	Ann. Intern. Med.	3	661	220	21.316
Archives of Physical Medicine and Rehabilitation	Arch. Phys. Med. Rehabil.	2	332	166	3.097
Anesthesia and Analgesia	Anesth. Analg.	1	187	187	4.307
Arthritis and Rheumatism	Arthritis Rheum.	1	171	171	NA
American Journal of Medicine	Am. J. Med.	1	161	161	4.528
Alternative Therapies in Health and Medicine	Altern. Ther. Health Med.	1	122	122	0.939
Acupuncture in Medicine	Acupunct. Med.	1	93	93	2.127
Autonomic Neuroscience: Basic & Clinical	Auton. Neurosci-Basic Clin.	1	67	67	2.2
British Journal of Sports Medicine	Br. J. Sports Med.	1	198	198	12.021
British Medical Journal	BMJ	2	310	155	30.22
BMC Musculoskeletal Disorders	BMC Musculoskelet. Disord.	1	186	186	1.878
British Journal of Anaesthesia	Br. J. Anaesth.	1	160	160	6.881
Bioelectromagnetics	Bioelectromagnetics	1	139	139	2.277
BMC Medicine	BMC Med.	1	115	115	6.783
British Dental Journal	Br. Dent. J.	1	88	88	1.306
Bulletin of the World Health Organization	Bull. World Health Organ.	1	81	81	6.962
BMC Complementary and Alternative Medicine	BMC Complement. Altern. Med.	1	68	68	2.831
Controlled Clinical Trials	Control Clin. Trials	1	425	425	NA
Cochrane Database of Systematic Reviews	Cochrane Database Syst. Rev.	20	2,360	118	7.893
Clinical Journal of Pain	Clin. J. Pain	1	66	66	2.891
Cephalalgia	Cephalalgia	1	133	133	4.867
Complementary Therapies in Medicine	Complement. Ther. Med.	2	192	96	2.064
Journal of Internal Medicine	J. Intern. Med.	2	397	199	6.874
Journal of the Royal Society of Medicine	J. R. Soc. Med.	1	202	202	5.239
Journal of Alternative and Complementary Medicine	J. Altern. Complement Med.	3	344	115	2.105
Journal of Clinical Oncology	J. Clin. Oncol.	1	163	163	32.959
Journal of Pain	J. Pain	1	146	146	4.62
JAMA Internal Medicine	JAMA Intern. Med.	1	140	140	18.654
Journal of Orthopaedic and Sports Physical Therapy	J. Orthop. Sports Phys. Ther.	2	202	101	3.839
Journal of Affective Disorders	J. Affect. Disord.	2	180	90	3.891
Journal of the American Board of Family Medicine	J. Am. Board Fam. Med.	1	109	109	2.663
The Journal of Family Practice	J. Fam. Pract.	1	100	100	0.693
journal of Neurology	J. Neurol.	1	84	84	3.958
Journal of Pediatric Hematology Oncology	J. Pediatr. Hematol. Oncol.	1	104	104	1.016
Pain	Pain	6	866	144	5.481
PLoS one	PLoS One	5	735	147	2.742
SPINE	SPINE	5	616	123	2.646
European Journal of Pain	Eur. J. Pain	3	444	148	3.49
Rheumatology	RHEUMATOLOGY	5	574	115	5.605
Evidence-Based Complementary and Alternative Medicine	Evid.-based Complement Altern. Med.	1	145	145	1.812
Stroke	Stroke	2	225	113	7.194
International Journal of Epidemiology	Int. J. Epidemiol.	1	106	106	7.708
International Journal of Obesity	Int. J. Obes.	1	89	89	NA
Osteoarthritis and Cartilage	Osteoarthritis Cartilage	1	82	82	4.79
Pediatrics	Pediatrics	1	80	80	5.359
Forsch Komplementarmed	Forsch. Komplement.med	1	73	73	NA
Sleep Medicine	Sleep Med.	1	71	71	3.035
World Journal of Gastroenterology	World J. Gastroenterol	1	66	66	3.669
Medicine (Baltimore)	Medicine (Baltimore)	1	65	65	1.552

## Data Availability

The original data used to support the findings of this study are included within the article.
